# Early-life exposure to air pollution and greater use of academic support services in childhood: a population-based cohort study of urban children

**DOI:** 10.1186/s12940-017-0210-z

**Published:** 2017-01-18

**Authors:** Jeanette A. Stingone, Katharine H. McVeigh, Luz Claudio

**Affiliations:** 1 Icahn School of Medicine at Mount Sinai, Department of Preventive Medicine, One Gustave Levy Pl, BOX 1057, New York, NY 10029 USA; 2 New York City Department of Health and Mental Hygiene, Division of Family and Child Health, 42-09 28th Street, Queens, NY 11101 USA

**Keywords:** Benzene, Early intervention, Special education, School outcomes, Traffic-related air pollution

## Abstract

**Background:**

There is a growing literature showing associations between prenatal and early-life exposure to air pollution and children’s neurodevelopment. However, it is unclear if decrements in neurodevelopment observed in epidemiologic research translate into observable functional outcomes in the broader pediatric population. The objective of this study was to examine the association between early-life exposures to common urban air toxics and the use of academic support services, such as early intervention and special education within a population-based cohort of urban children.

**Methods:**

Data for 201,559 children born between 1994 and 1998 in New York City were obtained through administrative data linkages between birth, early intervention and educational records. Use of academic support services was ascertained from birth through attendance in 3^rd^ grade. Census tract at birth was used to assign estimates of annual average ambient concentrations of benzene, toluene, ethylbenzene and xylenes (BTEX) using the 1996 National Air Toxics Assessment. Discrete-time hazard models were fit to the data and adjusted for confounders including maternal, childhood and neighborhood factors.

**Results:**

Children with higher exposures to BTEX compounds were more likely to receive academic support services later in childhood. For example, the adjusted hazard ratio comparing children exposed to the highest decile of benzene to those with lower exposure was 1.09 (95% confidence interval 1.05, 1.13). Results were consistent across individual BTEX compounds, for exposure metrics which summarized exposure to all four BTEX pollutants and for multiple sensitivity analyses.

**Conclusions:**

These findings suggest urban air pollution may affect children’s neurodevelopment and educational trajectories. They also demonstrate the use of public health data systems to advance children’s environmental health research.

**Electronic supplementary material:**

The online version of this article (doi:10.1186/s12940-017-0210-z) contains supplementary material, which is available to authorized users.

## Background

There is a growing literature showing that prenatal and early-life exposure to air pollution is associated with children’s neurodevelopment and behavior later in childhood [1–7]. Previous epidemiologic studies have demonstrated associations between greater prenatal or early-life exposure to ambient air pollutants, such as fine particulate matter and nitrogen dioxide, and lower IQ levels assessed in early childhood [3, 5, 6]. There is a need to determine if associations between air pollutants and children’s cognitive health exist beyond the localized birth cohorts of these studies and within the broader pediatric population. Additionally, it is unclear if decrements in neurodevelopment observed in epidemiologic research translate into observable functional outcomes in the population. Such outcomes could be measured as the increased need for developmental and academic support services, including early intervention programming and/or special education services. The use of academic support services is not only a marker of adverse neurodevelopment, but is also an early indicator of lower educational attainment later in life [8, 9].

Benzene, toluene, ethylbenzene and xylenes, collectively known as BTEX, are common urban air toxics that are naturally occurring in crude oil. They are among the most abundantly produced chemicals worldwide, and are emitted into the ambient air primarily from motor vehicle emissions and industrial uses of petroleum and petroleum-based products [10]. BTEX compounds can serve as a marker of traffic and point-source petroleum emissions in urban communities [11]. Previous research has found associations between maternal exposure to benzene during pregnancy and preterm birth [12], neural tube defects [13] and mental development among children whose mothers reported low intakes of fruits and vegetables [14]. However, other studies have found no association with ambient benzene exposure and measures of neurodevelopment [15].

The objective of this research is to examine the association between early-life exposure to BTEX compounds and the use of academic support services later in childhood. This research question was addressed by leveraging linked administrative data systems within the Longitudinal Study of Early Development (LSED) [16], a multi-system data linkage of pediatric records for New York City (NYC) children born between 1994 and 2004.

## Methods

The analysis of LSED was approved by the NYC Department of Health and Mental Hygiene Institutional Review Board (NYC DOHMH). This analysis was also approved by the Institutional Review Board of the Icahn School of Medicine at Mount Sinai.

### Study population

LSED is an administrative data linkage across five different public records systems: the birth and death registries, the lead poisoning prevention program, Early Intervention Program records, and educational data from the Department of Education [16]. Using these linked administrative records, we performed a retrospective cohort study to assess the relationship between early-life exposure to BTEX pollutants and the first use of academic support services in childhood. Children who were born in NYC to resident mothers from 1994 through 1998 and went on to attend third grade in a NYC public school by 2007 were eligible for inclusion within the study. Third grade educational records after 2007 were not included in the LSED data warehouse. Children with congenital anomalies documented on the birth record (*n* = 1,527) were excluded because they are more likely to have a greater need for academic support services in childhood [17]. Children who enrolled in third grade but did not have scores for third grade standardized tests were also excluded from this analysis (*n* = 4,902). Children who did not have scores for third grade standardized tests were more likely to be enrolled in schools designated for children with more severe disabilities and cognitive delays.

### Outcome assessment

Use of academic support services was defined as receipt of any early intervention programming (e.g. speech therapy, occupational therapy, etc.) as part of the NYC Early Intervention Program or enrollment in special education services within public school. The New York State Early Intervention Program was established in July of 1993 and provides services to children under 3 years of age with a confirmed disability or established developmental delay, in accordance with the Individual with Disabilities Education Act [18]. Services are covered by insurance and public funds, with no direct cost to the family. Children can be referred to the Early Intervention Program by a number of different sources, including hospitals, pediatricians, day care programs, local health units and community-based groups [19]. Children then undergo a multidisciplinary evaluation to determine eligibility for services, although some conditions (e.g. birthweight less than 1000 g) automatically qualify a child as eligible for services [19]. Once a child is deemed eligible, a service plan is created and children will then receive the specific programming outlined in their plan. Special education services, also provided in compliance with the Individual with Disabilities Education Act, covers a large range of services within the public educational system, including supplemental or modification of instruction within a general education environment, a separate special education classroom or school or home-based services for the severely disabled [20]. Similar to the Early Intervention Program, children must be determined eligible for services after an evaluation.

Children had records in LSED from the time of birth through attendance in third grade. The first documentation of receipt of academic support services within LSED was used to categorize children into the following outcome metric:

- Received early intervention services for the first time prior to 12 months of age
- Received early intervention services for the first time between 12 months and 24 months of age
- Received early intervention services for the first time between 24 and 36 months of age
- Did not receive early intervention services but received special education services for the first time in pre-kindergarten
- Did not receive early intervention services but received special education services for the first time in kindergarten
- Did not receive early intervention services but received special education services for the first time in either first, second or third-grade
- Never received any academic support services.

### Exposure assessment

Exposure to BTEX pollutants were assigned by linking LSED records to the EPA’s National Air Toxics Assessment (NATA) using the residential census tract listed on the birth record. NATA is a periodic assessment that uses emissions inventories and advanced modeling techniques to estimate annual average concentrations of air toxics at the census-tract level across the United States [21]. Because it was closest in time to the children’s birth years and included the individual BTEX components, the 1996 NATA assessment was used to assign exposure to children living in the 2,217 census tracts in existence in NYC during the 1990s. Estimated benzene concentrations from the 1996 NATA were assigned a higher overall certainty rating by EPA based on confidence in both emissions certainty and performance of the modeled estimates when compared to monitoring data [22]. The other BTEX pollutants were not evaluated for assignment of certainty ratings.

Due to the highly right-skewed distributions of the BTEX pollutants (see Additional file 1 Table S1), exposure to each pollutant was dichotomized at the 90^th^ centile. Children with estimated exposure greater than the 90^th^ centile were considered highly exposed to that pollutant, while children with exposure at or below the 90^th^ centile were considered the referent group. To examine the impact of exposure to multiple BTEX pollutants, a summary metric was constructed to compare children with high exposure to all four of the BTEX pollutants to children who had lower exposure to at least one of these pollutants. Additionally, a second summary metric was constructed to compare children with high exposure to at least one BTEX pollutant to children who had lower exposure to all BTEX pollutants.

### Identification of confounders

Review of the existing literature for factors that may affect this outcome and/or exposure and directed acyclic graph analysis were used to identify potential confounders to include in multivariable models [23]. The identified confounders were: maternal race/ethnicity, maternal nativity, maternal educational attainment, maternal age at delivery, maternal marital status at the time of delivery, maternal insurance coverage at delivery, child’s maximum blood lead level and a neighborhood deprivation index [24], derived from census variables. Assigned using the census tract of the residential address reported on the birth record, the neighborhood deprivation index was calculated as the first component resulting from a principal component analysis of the following eight variables from the 2000 US Census: percent of residents with college degree, percent unemployment, percent of residents with a management/professional occupation, percent residential crowding, percent below 200% of the federal poverty line, percent of households receiving public assistance, percent of residents of nonwhite race and percent of households that were linguistically isolated. Maternal race/ethnicity was reported by the mother on the birth certificate. The investigators used four racial/ethnic classifications: Black, non-Latina, White, non-Latina, Latina, and Asian/Other Race. Approximately 22% (*n* = 44,317) of children were missing measurements of early-childhood blood lead levels. Single-chain Markov chain Monte Carlo methodology [25] was used to impute missing blood lead values. The imputation model included the following variables: ambient BTEX concentration estimates, maternal race/ethnicity, maternal nativity, maternal educational attainment, maternal age at delivery, maternal marital status at the time of delivery, maternal insurance coverage at delivery, neighborhood deprivation index, use of academic support services, child’s sex, year that housing was built, borough of residence, mother’s employment status and mother’s participation in the Women, Infants and Children supplementary nutrition program. A total of five imputations were run.

### Statistical analysis

Discrete-time proportional hazard models [26] were constructed in order to assess the relationship between the exposure metrics described above and first-use of academic support services. These models were constructed using logistic regression fit with a complementary log-log function. This allows for the estimation of the continuous proportional hazards and is invariant to the length of the interval. The baseline hazard was modeled with indicator terms for each time period. As a result, the estimation of the hazards is also invariant to the longer length of time represented by the last time category, as long as the proportional hazards assumption was met [26]. The proportional hazards assumption was tested using an a-priori alpha level of 0.01, due to the very large sample size. Both crude and adjusted hazard ratios (HRs) and 95% confidence intervals (CI) were obtained. Results were obtained for each of the five imputations and then pooled using Rubin’s rules to account for the variation in imputed values across the different imputations [25]. Analyses were repeated after restricting to children who lived in the same census-tract from birth through third grade, removing from the analysis the potential bias related to residential mobility during childhood. Data were stratified by maternal race and child sex, and analyses were repeated in order to assess if associations between BTEX and use of academic services were consistent across levels of these factors. Because this study assigns exposure using a single annual estimate from 1996, we repeated our analysis after stratifying by the child’s birth year, to assess the impact of exposure misclassification due to the lack of temporal exposure data.

## Results

Demographic and exposure characteristics are provided in Table 1. This study population displays the racial/ethnic and educational diversity reflective of NYC. Forty percent of mothers identified as Latina, while another 31.9% were Black, non-Latina. Slightly fewer than half of all mothers were born in the U.S. Children with high exposure to at least one pollutant were 14.8% of the total population. Slightly more than half of children with high exposure to at least one pollutant had high exposure to all pollutants. Children born in areas with high BTEX compounds were more likely to be born to mothers who were Latina. Their mothers were also more likely to have either less than a high school education or more than a college education than mothers of children with lower pollutant exposures. Exposed children were less likely to have elevated blood lead levels compared to children who weren’t highly exposed to BTEX pollutants.

**Table 1:** Demographic and Exposure Characteristics of the New York City Longitudinal Study of Early Development, 1994–1998

	Total Population (*N* = 201,559)	BTEX greater than 90^th^ centile for at least one pollutant (*N* = 26010)	BTEX lower than 90^th^ centile for all pollutants (*N* = 175549)	BTEX greater than 90^th^ centile for all pollutants (*N* = 15514)	BTEX lower than 90^th^ centile for at least one pollutant (*N* = 186045)
Demographic Factor	*N* (%)	*N* (%)	*N* (%)	*N* (%)	*N* (%)
Maternal Race					
White, non-Latina	32730 (16.2)	3973 (15.3)	28757 (16.4)	2415 (15.6)	30315 (16.3)
Black, non-Latina	64353 (31.9)	4309 (16.6)	60044 (34.2)	2611 (16.3)	61742 (33.2)
Latina	81716 (40.5)	14930 (57.4)	66786 (38.0)	8666 (55.6)	73050 (36.2)
Asian/Other	22760 (11.3)	2798 (10.8)	19962 (11.4)	1822 (11.7)	20938 (11.3)
Maternal Nativity					
Born in the U.S.	97874 (48.6)	12140 (46.7)	85734 (48.8)	7258 (46.8)	90616 (48.7)
Maternal Age, years mean (sd)	27.2 (6.4)	27.3 (6.7)	27.2 (6.4)	27.6 (6.8)	27.2 (6.4)
Maternal Education					
0–11 years	69863 (34.7)	9775 (37.6)	60088 (34.2)	5730 (36.9)	64133 (34.5)
12 years	77735 (38.6)	8936 (34.4)	68799 (39.2)	5094 (32.8)	72641 (39.0)
13–15 years	32900 (16.3)	3688 (14.2)	29212 (16.6)	2206 (14.2)	30694 (16.5)
16 or more years	20161 (10.4)	3611 (13.9)	17450 (9.9)	2484 (16.0)	18577 (10.0)
Maternal Insurance at time of Delivery					
Medicaid	131295 (65.1)	18722 (72.0)	112573 (64.1)	11130 (71.7)	120165 (64.6)
HMO	18728 (9.3)	2106 (8.1)	16622 (9.5)	1321 (8.5)	17407 (9.4)
Other 3rd Party	41768 (20.7)	4460 (17.1)	37308 (21.3)	2715 (17.5)	39053 (21.0)
Self-Paid	9768 (4.8)	722 (2.8)	9046 (5.2)	348 (2.3)	9420 (5.1)
Maternal Marital Status					
Married	83693 (41.5)	10210 (39.3)	73483 (41.9)	6140 (39.6)	77553 (41.7)
Maximum Venous Blood Lead (μg/dL)^a^					
Less than 3	33716 (16.7)	4790 (18.4)	28926 (16.5)	3108 (20.0)	30608 (16.5)
3- < 5	84289 (41.8)	10784 (41.5)	73505 (41.9)	6364 (41.0)	77925 (41.9)
5- < 10	71485 (35.5)	9063 (34.8)	62422 (35.6)	5298 (34.1)	66187 (35.6)
10 and greater	12069 (6.0)	1374 (5.3)	10695 (6.1)	744 (4.8)	11325 (6.1)

^a^Average of five imputations

Crude counts of incident cases of children who received academic support services across time, along with the population-at-risk, are presented in Table 2. Overall, 18.3% of children received academic support services sometime between birth and completion of third grade. In addition to the overall study population, incident cases are shown stratified by their exposure to BTEX pollutants. Children with greater exposure to either one or multiple BTEX pollutants were more likely to use academic support services than those children with lower levels of exposure. Among children receiving early intervention services, 91% received speech therapy or special instruction services, either alone, in combination with each other, or in combination with physical and/or occupational therapy.

**Table 2:** Population at-risk and incident cases of children who received academic support services across time by exposure to benzene, toluene, ethyl benzene and xylene pollutants, New York City Longitudinal Study of Early Development 1994–1998

		Full Population	Population with BTEX greater than 90^th^ centile for at least one pollutant	Population with BTEX lower than 90^th^ centile for all pollutants	Population with BTEX greater than 90^th^ centile for all pollutants	Population with BTEX lower than 90^th^ centile for at least one pollutant
Time Point	Population at-Risk	Children receiving academic support services for the first time	Children receiving academic support services for the first time	Children receiving academic support services for the first time	Children receiving academic support services for the first time	Children receiving academic support services for the first time
Less than 12 months of age	201559	1004	121	883	77	927
12 months–24 months of age	200555	2599	339	2260	200	2399
24 months–36 months of age	197956	7962	1128	6834	673	7289
Pre-School Special Education	189994	5445	801	4644	460	4985
Kindergarten Special Education	184549	2987	388	2599	208	2779
3rd grade Special Education	181562	16881	2434	14447	1432	15449

**Fig. 1:**
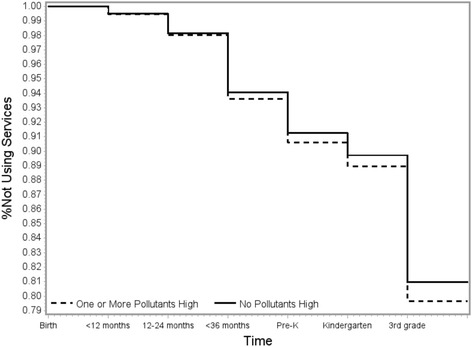
Proportion of children not using academic support services, stratified by high exposure to at least one BTEX pollutant. New York City Longitudinal Study of Early Development 1994–1998 birth cohorts. Predicted probability curves resulting from adjusted discrete hazard models of time to using academic support services. Covariates fixed at a married White, non-Latina mother, who was born in the US, completed high school, delivered the child at 25, had Medicaid at the time of delivery, who was living in a community with the mean neighborhood deprivation index and whose child had a childhood blood lead less than 3 micrograms/deciliter

**Fig. 2:**
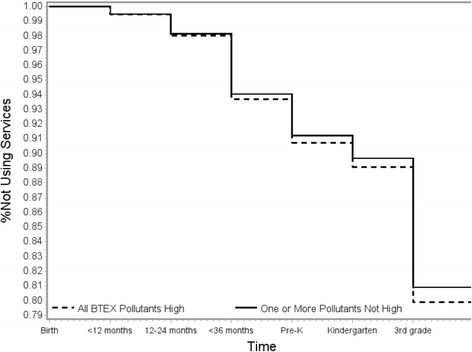
Proportion of children not using academic support services, stratified by high exposure to all BTEX pollutants. New York City Longitudinal Study of Early Development 1994–1998 birth cohorts. Predicted probability curves resulting from adjusted discrete hazard models of time to using academic support services. Covariates fixed at a married White, non-Latina mother who was born in the US, completed high school, delivered the child at 25, had Medicaid at the time of delivery, who was living in a community with the mean neighborhood deprivation index and whose child had a childhood blood lead less than 3 micrograms/deciliter

Results in Table 3 show that children with high exposure to BTEX pollutants have a slightly greater hazard of receiving academic support services than children who were born in areas with lower levels of these pollutants. Adjusted HRs were slightly closer to the null than crude estimates. There was no evidence of violations of the proportional hazards assumption. These results are consistent across all pollutants and for the two summary metrics. Estimated HRs were slightly greater among the population of children who lived in the same census-tract from birth through third grade. Figures 1 and 2 show fitted survival curves when looking at the summary exposure metrics and use of academic support services in the full population. On both figures, the difference between the exposed and unexposed starts to become visible between 24 and 36 months of age. The greatest initiation of academic support services, regardless of exposure status, occurs between Kindergarten and third grade. We observed no considerable differences in results when stratifying by child’s sex or maternal race/ethnicity; however HRs were lower for children born in 1994 and 1998, the two years furthest from 1996, the year that exposure was modeled using NATA (see Additional file 1, Tables S2–S4).

**Table 3:** Associations^a^ between exposure to BTEX pollutants and first use of academic support services, New York City Longitudinal Study of Early Development 1994–1998

Pollutant	Crude HR	95% CI	Adjusted^b^ HR	95% CI	Non-movers Adjusted^b^ HR	95% CI
	*N* = 201559		*N* = 201559		*N* = 57025	
Benzene greater than 90^th^ centile	1.13	1.10, 1.17	1.09	1.05, 1.13	1.10	1.04, 1.17
Benzene at or lower than 90^th^ centile	1		1		1	
Toluene greater than 90^th^ centile	1.09	1.05, 1.12	1.06	1.02, 1.09	1.08	1.02, 1.15
Toluene at or lower than 90^th^ centile	1		1		1	
Ethylbenzene greater than 90^th^ centile	1.10	1.07, 1.14	1.06	1.03, 1.10	1.10	1.03, 1.16
Ethylbenzene at or lower than 90^th^ centile	1		1		1	
Xylenes greater than 90^th^ centile	1.10	1.07, 1.14	1.07	1.03, 1.10	1.10	1.04, 1.17
Xylenes at or lower than 90^th^ centile	1		1		1	
BTEX greater than 90^th^ centile for at least one pollutant	1.12	1.09, 1.15	1.08	1.05, 1.11	1.11	1.05, 1.17
BTEX at or lower than 90^th^ centile for all pollutants	1		1		1	
BTEX greater than 90^th^ centile for all pollutants	1.08	1.05, 1.13	1.06	1.02, 1.10	1.09	1.02, 1.16
BTEX at or lower than 90^th^ centile for at least one pollutant	1		1		1	

^a^Crude and adjusted hazard ratios and 95% confidence intervals resulting from discrete hazard models with complementary log-log function

^b^Adjusted for the following confounders maternal race/ethnicity, maternal nativity, maternal educational attainment, maternal age at delivery, maternal marital status at the time of delivery, maternal insurance coverage at delivery, child’s maximum blood lead level and a neighborhood deprivation index [24], derived from census variables

## Discussion

This study is one of the first population-based studies to observe an association between higher early-life exposure to BTEX compounds and greater use of academic support services among a large cohort of urban children. After adjusting for a number of confounders, elevated HRs for use of academic support services were relatively small in magnitude but consistently observed across exposure metrics for all individual BTEX compounds, as well as summary metrics that indicated high exposure to at least one BTEX compound and high exposure to all BTEX compounds. HRs were slightly larger in magnitude when the population was restricted to children who lived in the same census-tract from birth through third grade, suggesting that results were not due to bias related to residential mobility during childhood. Results did not change when excluding children with imputed blood lead measurements. For example, when estimating the hazard ratio of use of academic support services associated with high benzene exposure after excluding children with imputed blood lead levels, the HR was 1.09 (95% CI 1.06, 1.13).

These findings add to a growing literature showing that urban air pollution can impact children’s neurodevelopment and cognitive health [1]. Previous work in NYC and other urban areas have found associations between prenatal and/or early-life exposure to various air pollutants and IQ [3, 7], as well other measures of neurodevelopment, including attention, behavior and memory scores [6]. These studies have been performed in smaller birth cohorts, and it was previously unclear how these decrements in neurodevelopment would translate into children’s later school outcomes and whether these results were generalizable to the broader population of urban children. Using modeled exposure data and existing administrative resources for a large, representative population of over 200,000 urban children, this study supports the findings of previous research and documents an association between greater exposure to common urban air pollutants and greater use of academic support services, such as early intervention programming and special education services.

The majority of children receiving early intervention services received speech therapy and/or special instruction services. This could suggest that most children receiving services have learning, cognitive or socio-behavioral delays, as opposed to purely physical delays that require intervention such as physical or occupational therapy to improve gross and fine motor skills. A recent systematic review highlighted a number of studies which found associations between prenatal and early-life air pollutant exposure and various measures of neurodevelopment in children [1]. The authors pointed to oxidative stress as a potential mechanism between air pollution exposure and adverse neurodevelopment. Other studies have observed that exposure to fine particulate matter was associated with global DNA hypomethylation, suggesting epigenetic mechanisms could play a role in the potential neurotoxicity of air pollutants [27]. More detailed information on the types of interventions and services children received, as well as the types of developmental delays contributing to their use of services, would help inform hypothesized mechanisms and suggest further avenues of research.

There are some limitations of our study. The BTEX exposures estimated in this study are the annual average estimate of pollutant concentrations in 1996 for each census tract in NYC. This lack of temporal variability introduces exposure misclassification, and as demonstrated in our results, HRs were larger in magnitude for children born closer in time to 1996 (i.e., the children whose exposure may be better represented by 1996 estimates). This could suggest that exposure misclassification causes our effect estimates to underrepresent the true hazard of BTEX exposure. The magnitude of the HRs was relatively small, although they are consistent across the different BTEX compounds and summary metrics. There are additional sources of non-differential exposure misclassification within the study that could also contribute to the relatively small magnitude of the effect estimate. Both a lack of information on time spent in other areas and lack of data on residential mobility during pregnancy would introduce non-differential misclassification.

We were able to conduct a sensitivity analysis to restrict to children who never moved from their census-tract at birth, removing the potential for residential mobility during childhood to impact our results, and observed slightly larger effect estimates for the HRs. This could suggest that exposure misclassification within the study causes the results to underestimate the true hazard. It could also suggest that cumulative exposure during early childhood could have a larger impact on a child’s neurodevelopment and academic outcomes than exposures limited to the time around birth. Future research which compares children who remain in high-pollution neighborhoods throughout their childhood to children who move to lower pollution neighborhoods would help elucidate the question of exposure timing. Additionally, recent research has observed cross-sectional associations between parent-reported grade point averages and children’s ambient exposure to air toxics at school [28]. Incorporating exposure at school locations in future research would also improve our ability to estimate the effect of air toxics on children’s cognitive health.

There are documented differences in academic support service utilization by racial and socioeconomic factors in NYC and other urban areas [29]. However, associations between BTEX compounds and use of academic support services were observed for all racial groups in stratified analyses, even after adjusting for maternal education, insurance status and neighborhood measures of socioeconomic status. This suggests that differences in utilization based on demographic factors would not solely be driving the results observed in this study.

In NYC, motor vehicles are the primary source of BTEX compounds, with off-road gasoline engines, petroleum transport/storage and solvent usage also contributing to estimated ambient concentrations of these pollutants [11]. Within the 1996 NATA, benzene is the marker of traffic and motor vehicle exhaust that is estimated with the highest level of confidence. However, motor vehicle exhaust and traffic produce a number of other pollutants that are highly correlated to the gaseous BTEX compounds because of the common source. Fine particulate matter, polycyclic aromatic hydrocarbons and other contaminants stemming from exhaust have been shown in previous research to be associated with children’s neurodevelopment [1] and could underlie the results observed in this study. Further research could collect more detailed environmental data on the multiple exposures stemming from traffic and motor vehicle exhaust, including noise, in order to determine if BTEX compounds are the causal contributor to the association with greater use of academic support services.

Additionally, other environmental contaminants, from either air or other exposure routes, could be associated with both BTEX exposure and use of services. Because NATA estimates ambient concentrations of multiple air toxics, it was possible to assess the potential for a non-traffic related air toxic to contribute to our results. Perchloroethylene is a solvent, most commonly associated with the dry-cleaning industry, and is common in the urban environment. Including perchloroethylene as a covariate in the model between benzene exposure and use of academic support services changed the effect estimate by less than 10% (HR 1.08, 95%CI 1.04, 1.12). However, this is only one of many potential environmental contaminants that could contribute to unmeasured confounding. This study was also able to adjust for blood lead, but exposure to other urban environmental contaminants, including those not estimated by NATA such as pesticides, have been shown to be associated with neurodevelopment [30], and may be confounding the results.

The study’s generalizability is limited to children who were born in NYC and eventually enrolled in NYC public school. It is possible that children who go on to enroll in private school or whose families move out of NYC, may have different exposure profiles and utilization patterns for academic support services. Due to the exclusion criteria applied within our analysis, these results may not be generalizable to children with congenital anomalies and children with more severe forms of cognitive delays that did not sit for the statewide standardized tests.

## Conclusions

Despite these potential limitations, these findings suggest an association between ambient exposure to BTEX and use of academic support services later in childhood. In 2010, NYC early intervention services reached a total cost of $482.3 million, with approximately $116 million not covered by insurance and therefore absorbed into public budgets [31]. Special education services are also a large component of the educational budget, with the average special education student costing $29,911 in New York State, compared to $11,256 to educate a general education student [32]. Determining if ambient air pollution increases the use of these resource-intensive services has important public policy implications for urban areas. Additionally, children who require developmental and academic support services such as special education are more likely to have poorer educational outcomes including failing to complete high school [8, 9], and educational attainment is a recognized social determinant of health among adults [33, 34].

Although the specific delays and diagnoses contributing to children’s use of academic support services are unknown, it is known that special instruction services within the Early Intervention Program are often provided to children with social and behavioral problems. Previous research has observed associations between prenatal or early-life exposure to traffic-related air pollutants and adverse measures of childhood behavior [6, 35]. This could suggest that greater exposure to air pollutants in the urban environment can affect children’s behavioral development, potentially leading to adverse outcomes and greater need of mental health services later in adulthood [36, 37]. Longitudinal research designs are needed to assess the hypothesis that early-life exposure to air toxics alters children’s educational trajectories and contribute to adverse physical and mental health outcomes later in life.

This study also demonstrates the potential for public health data systems to contribute to research on the broader pediatric effects of environmental pollution. Linking and integrating information systems to improve children’s health has recently become a focus for improving healthcare and public health service delivery [38]. Within this study, we were able to leverage a comprehensive data linkage in order to construct a large, retrospective cohort of children with environmental and educational data available at multiple time-points in childhood. These types of studies can efficiently address important research questions within the field of children’s environmental health, as well as inform more targeted studies of environmental health.

In summary, this research adds to the growing literature that urban air pollution can affect the neurodevelopment and educational outcomes of children. Future work will be needed to determine if BTEX compounds are driving these associations, or are a marker for the mixture of exposures stemming from vehicle exhaust and traffic. Additionally, more refined measures of both exposure and outcome are needed to reduce misclassification impacts on results in order to assess the true magnitude of these exposures on children’s health.

## Additional file

Additional file 1: The file contains 4 supplemental tables containing additional demographic characteristics and results of sensitivity analyses referenced in the main manuscript text. **Table S1.** Additional demographic and exposure characteristics of the New York City Longitudinal Study of Early Development, 1994–1998 birth cohort. **Table S2.** Adjusted^1^ hazard ratios and 95% confidence intervals describing the relationship between exposure to benzene and first use of academic support services, stratified by maternal race. NYC Longitudinal Study of Early Development 1994–1998 birth cohort. **Table S3.** Adjusted^1^ hazard ratios and 95% confidence intervals describing the relationship between exposure to benzene and first use of academic support services, stratified by child sex. NYC Longitudinal Study of Early Development 1994–1998 birth cohort. **Table S4.** Adjusted^1^ hazard ratios and 95% confidence intervals describing the relationship between exposure to benzene and first use of academic support services, stratified by child’s year of birth. NYC Longitudinal Study of Early Development 1994–1998 birth cohort. (DOCX 24 kb)

## Abbreviations

BTEX: Benzene, toluene, ethyl benzene and xylenes
CI: Confidence interval
DOHMH: Department of health and mental hygiene
HR: Hazard ratio
LSED: Longitudinal study of early development
NATA: National air toxics assessment
NYC: New York city
