# Supplementary material for: Early-life exposure to air pollution and greater use of academic support services in childhood: a population-based cohort study of urban children
Source: Environ Health. 2017 Jan 18;16:2. doi: 10.1186/s12940-017-0210-z (PMC5241986; doi:10.1186/s12940-017-0210-z)
Supplement: Additional file 1: — The file contains 4 supplemental tables containing additional demographic characteristics and results of sensitivity analyses referenced in the main manuscript text. Table S1. Additional demographic and exposure characteristics of the New York City Longitudinal Study of Early Development, 1994–1998 birth cohort. Table S2. Adjusted1 hazard ratios and 95% confidence intervals describing the relationship between exposure to benzene and first use of academic support services, stratified by maternal race. NYC Longitudinal Study of Early Development 1994–1998 birth cohort. Table S3. Adjusted1 hazard ratios and 95% confidence intervals describing the relationship between exposure to benzene and first use of academic support services, stratified by child sex. NYC Longitudinal Study of Early Development 1994–1998 birth cohort. Table S4. Adjusted1 hazard ratios and 95% confidence intervals describing the relationship between exposure to benzene and first use of academic support services, stratified by child’s year of birth. NYC Longitudinal Study of Early Development 1994–1998 birth cohort. (DOCX 24 kb) [file 12940_2017_210_MOESM1_ESM.docx]

**Supplemental Material**

**Title:** Early-life exposure to air pollution and greater use of academic support services in childhood: a population-based cohort study of urban children

| Table S1: Additional demographic and exposure characteristics of the New York City Longitudinal Study of Early Development, 1994-1998 birth cohort | | | | | |
| --- | --- | --- | --- | --- | --- |
|  | Total Population (N=201,559) | BTEX greater than 90^th^ centile for at least one pollutant  (N=26010) | BTEX lower than 90^th^ centile for all pollutants  (N=175549) | BTEX greater than 90^th^ centile for all pollutants  (N=15514) | BTEX lower than 90^th^ centile for at least one pollutant  (N=186045) |
| Demographic Factor | N (%) | N(%) | N (%) | N (%) | N (%) |
| Maternal Borough of Residence at Time of Delivery | | | | | |
| Brooklyn | 71076 (35.3) | 3494 (13.4) | 67582 (38.5) | 13 (0.1) | 71063 (38.2) |
| Bronx | 41540 (20.6) | 2216 (8.5) | 39324 (22.4) | 603 (3.9) | 40937 (22.0) |
| Manhattan | 30137 (15.0) | 18923 (72.8) | 11214 (6.4) | 14857 (95.8) | 15280 (8.2) |
| Queens | 48503 (24.1) | 1377 (5.3) | 47126 (26.8) | 41 (0.3) | 48462 (26.0) |
| Staten Island | 10303 (5.1) | 0 (0) | 10303 (5.1) | 0 (0) | 10303 (5.5) |
|  |  |  |  |  |  |
| Child's Sex |  |  |  |  |  |
| Male | 100505 (49.9) | 12984 (49.9) | 87521 (49.9) | 7735 (49.9) | 92770 (49.9) |
| Female | 101054 (50.1) | 13026 (50.1) | 88028 (50.1) | 7779 (50.1) | 93275 (50.1) |
|  |  |  |  |  |  |
| Child Eligible for Free Lunch | | | | | |
| Yes | 172155 (85.4) | 21861 (84.0) | 150294 (85.6) | 12808 (82.6) | 159347 (85.6) |
|  |  |  |  |  |  |
| Benzene, median (min, 90th centile, maximum) | 3.1 (1.4, 4.9, 13.8) | 5.6 (3.1, 7.8, 13.8) | 3.1 (1.4, 3.7, 4.8) | 5.9 (4.9, 8.4, 13.8) | 3.1 (1.4, 4.0, 8.0) |
| Toluene, median (min, 90th centile, maximum) | 11.1 (4.3, 16.2, 105) | 18.2 (11.4, 23.7, 105) | 10.7 (4.3, 13.6, 16.1) | 20.8 (16.2, 27.5, 36.2) | 10.9 (4.3, 14.4, 105) |
| Ethylbenzene, median (min, 90th centile, maximum) | 1.6 (0.6, 2.6, 6.8) | 3.0 (1.4, 4.2, 6.8) | 1.5 (0.6, 2.0, 2.6) | 3.7 (2.7, 4.8, 6.8) | 1.5 (0.6, 2.1, 2.9) |
| Xylene, median (min, 90th centile, maximum) | 6.6 (2.6, 10.6, 29.0) | 12.7 (5.9, 17.6, 29.0) | 6.3 (2.6, 8.2, 10.6) | 15.3 (10.8, 20.0, 29.0) | 6.4 (2.6, 8.9, 18.9) |

.

| Table S2: Adjusted^1^ hazard ratios and 95% confidence intervals describing the relationship between exposure to benzene and first use of academic support services, stratified by maternal race. NYC Longitudinal Study of Early Development 1994-1998 birth cohort. | | | | | | | | |
| --- | --- | --- | --- | --- | --- | --- | --- | --- |
|  | Asian/Other Race | | Black, non-Latina | | Latina | | White, non-Latina | |
| Pollutant | Adjusted HR | 95% CI | Adjusted HR | 95% CI | Adjusted HR | 95% CI | Adjusted HR | 95% CI |
|  | N=22760 |  | N=64353 |  | N=81716 |  | N=32730 |  |
| Exposure to highest decile of benzene | 1.07 | 1.03, 1.21 | 1.08 | 1.02, 1.15 | 1.12 | 1.03, 1.21 | 1.14 | 0.98, 1.32 |
| Less than highest decile of benzene | 1 |  | 1 |  | 1 |  | 1 |  |

| Table S3: Adjusted^1^ hazard ratios and 95% confidence intervals describing the relationship between exposure to benzene and first use of academic support services, stratified by child sex. NYC Longitudinal Study of Early Development 1994-1998 birth cohort. | | | | |
| --- | --- | --- | --- | --- |
|  | Female Children | | Male Children | |
|  | Adjusted HR | 95% CI | Adjusted HR | 95% CI |
| Pollutant | N=101054 |  | N=100505 |  |
| Exposure to highest decile of benzene | 1.10 | 1.05, 1.14 | 1.09 | 1.05, 1.13 |
| Less than highest decile of benzene | 1 |  | 1 |  |

^1^Adjusted for the following confounders maternal race/ethnicity, maternal nativity, maternal educational attainment, maternal age at delivery, maternal marital status at the time of delivery, maternal insurance coverage at delivery, child’s maximum blood lead level and a neighborhood deprivation index, derived from census variables.

^1^Adjusted for the following confounders maternal race/ethnicity, maternal nativity, maternal educational attainment, maternal age at delivery, maternal marital status at the time of delivery, maternal insurance coverage at delivery, child’s maximum blood lead level and a neighborhood deprivation index, derived from census variables.

^1^Adjusted for the following confounders maternal race/ethnicity, maternal nativity, maternal educational attainment, maternal age at delivery, maternal marital status at the time of delivery, maternal insurance coverage at delivery, child’s maximum blood lead level and a neighborhood deprivation index, derived from census variables.

| Table S4: Adjusted^1^ hazard ratios and 95% confidence intervals describing the relationship between exposure to benzene and first use of academic support services, stratified by child’s year of birth. NYC Longitudinal Study of Early Development 1994-1998 birth cohort. | | | | | | | | | | |
| --- | --- | --- | --- | --- | --- | --- | --- | --- | --- | --- |
|  | 1994 | | 1995 | | 1996 | | 1997 | | 1998 | |
| Pollutant | Adjusted HR | 95% CI | Adjusted HR | 95% CI | Adjusted HR | 95% CI | Adjusted HR | 95% CI | Adjusted HR | 95% CI |
|  | N=38278 |  | N=38635 |  | N=41527 |  | N=42748 |  | N=40371 |  |
| Exposure to highest decile of benzene | 1.04 | 0.96, 1.13 | 1.14 | 1.06, 1.23 | 1.09 | 1.02, 1.17 | 1.12 | 1.05, 1.20 | 1.06 | 0.98, 1.14 |
| Less than highest decile of benzene | 1 |  | 1 |  | 1 |  | 1 |  | 1 |  |
